# Exploring patients’ views regarding the support and rehabilitation needs of people living with myocardial ischaemia and no obstructive coronary arteries: a qualitative interview study

**DOI:** 10.1136/bmjopen-2024-086770

**Published:** 2024-12-10

**Authors:** Helen Humphreys, Danielle Paddock, Sarah Brown, Aynsley Cowie, Colin Berry, Susan Dawkes, Simon Nichols

**Affiliations:** 1 Centre for Behavioural Science and Applied Psychology, Sheffield Hallam University, Sheffield, UK; 2 International Heart Spasms Alliance, London, UK; 3 Cardiovascular Care Partnership UK, London, UK; 4 Cardiac Rehabilitation, NHS Ayrshire and Arran, Kilmarnock, UK; 5 BHF Glasgow Cardiovascular Research Centre, University of Glasgow, Glasgow, UK; 6 Edinburgh Napier University, Edinburgh, UK; 7 School of Nursing and Midwifery, Edith Cowan University, Joondalup, Perth, Australia

**Keywords:** Coronary heart disease, Ischaemic heart disease, QUALITATIVE RESEARCH, REHABILITATION MEDICINE, Cardiovascular Disease

## Abstract

**Abstract:**

**Objectives:**

This study aimed to generate new qualitative insights to understand the rehabilitation needs of people living with a confirmed or presumed diagnosis of ischaemia with no obstructive coronary arteries (INOCA), explore which aspects of current cardiovascular prevention and rehabilitation programmes could meet the needs of people with INOCA and where adjustments (if any) may be appropriate.

**Design:**

Semistructured qualitative interview study.

**Participants:**

Interviews were undertaken (n=17; 88% female, age range 31–69 years) with people with a confirmed or presumed diagnosis of INOCA.

**Results:**

Findings highlighted concerns around a lack of evidence-based guidance for cardiovascular prevention and rehabilitation programmes for patients with INOCA. Participants expressed a desire for modular cardiovascular prevention and rehabilitation programmes that could be accessed flexibly to accommodate episodic fluctuations in symptoms. Participants suggested that existing cardiovascular prevention and rehabilitation programme content needed adjustment including enhanced psychosocial support, supervised low-impact physical activity and specialist dietary advice and medication reviews. Additional elements specific to INOCA should be made available as appropriate including acute care planning and a module to provide information and support for female-specific issues. The importance of involving INOCA patients in the codesign of future programmes and associated training was emphasised.

**Conclusions:**

People with INOCA are willing to engage with cardiovascular prevention and rehabilitation programmes and express a desire for more support. This patient group shares some barriers to rehabilitation programme attendance with other cardiac patient groups, but they also have specific concerns about the need for improved professional knowledge and evidence-based guidance regarding the management of INOCA. Cardiovascular rehabilitation programmes need to be delivered flexibly and individually tailored to ensure the relapsing and remitting nature of INOCA and associated support needs are addressed.

**STRENGTHS AND LIMITATIONS OF THIS STUDY**

- Qualitative interviews enabled an in-depth enquiry into the support and rehabilitation needs of people living with ischaemia with no obstructive coronary arteries.
- Inductive thematic analysis ensured descriptions and interpretations of the views of participants were grounded in the data.
- The sample included a mix of patients who had previously attended and those who had never attended cardiac rehabilitation.
- Participants were recruited primarily from online patient support forums and thus findings may not represent the views of digitally excluded patients or those not currently engaged in patient networks.
- Information about sociodemographic status and length of time since diagnosis were not collected, limiting conclusions about how these aspects might influence patients’ views.

## Introduction

 Ischaemia with no obstructive coronary arteries (INOCA) is an umbrella term for a range of conditions with different underlying pathologies including endothelial dysfunction, microvascular remodelling, microvascular and epicardial spasm and vasomotor abnormalities.^1^ These conditions affect up to two in five patients who present with chest pain.^2^ In contrast to obstructive coronary artery disease (CAD), where men are more likely to be affected, INOCA disproportionally affects women, particularly between the ages of 45–65.^1 3^ INOCA patients have been found to have more limiting dyspnoea, a comparable angina burden but reduced quality of life compared with patients with obstructive CAD.^4^

Approximately 30% of INOCA patients report symptoms of depression and approximately 60% of patients experience chest pain.^5^ People with INOCA typically have a low peak oxygen uptake, resulting in limited functional independence,^6^ and have elevated risk of major adverse cardiovascular events and mortality.^7^ There is a clear need to develop disease-modifying treatments for microvascular and vasospastic disease in patients with INOCA.

Differences in exercise capacity and responses to medication suggest that individualised, bespoke treatments are required. The CorMicA trial found that interventional diagnostic procedures with targeted drug therapy (stratified medical therapy) led to improvements in symptoms of angina and quality of life.^3^ For some people with INOCA, stratified medical therapy included a cardiovascular prevention and rehabilitation programme (CPRP; henceforth referred to as ‘cardiac rehabilitation’).^3^

Comprehensive cardiac rehabilitation typically includes lifestyle and risk factor management which includes exercise, diet and weight management interventions, smoking cessation, health behaviour change and education, psychosocial support and medical management.^8 9^ In the UK, the British Association for Cardiovascular Prevention and Rehabilitation specify which groups of people should be offered cardiac rehabilitation. Until 2023, INOCA patients were not included as the evidence of benefit was not known.^9 10^ Updated guidelines now recommend that people with INOCA are offered cardiac rehabilitation.^8^ However, the evidence regarding benefits remains inconclusive, and no qualitative research has previously been undertaken regarding patients’ rehabilitation needs. A crucial early step in developing appropriate, comprehensive and effective cardiac rehabilitation is to understand what those needs are and the extent to which cardiac rehabilitation in its current format can meet them.

This was an exploratory, inductive qualitative study designed to investigate the views of patients with INOCA towards cardiac rehabilitation. An inductive approach is recommended when there is little existing knowledge and the study is not based on existing theoretical frameworks or hypotheses. However, existing British Assocation for Cardiaovascular Prevention and Rehabilitation standards^8^ were used to inform the interview guide, encouraging exploration of views across all aspects of comprehensive cardiac rehabilitation content. Research questions for the study were:

1. Understand the support and rehabilitation needs of people living with a confirmed or presumed diagnosis of INOCA.
2. Explore which aspects of current cardiac rehabilitation could meet the needs of people with INOCA and where adjustments (if any) may be appropriate.

A secondary aim of the study was to explore the broader lived experiences of this patient group beyond cardiac rehabilitation. Participants’ accounts relating to other aspects of their lived experience generated a separate set of themes and are reported separately.^11^

## Method

### Recruitment

Semistructured interviews were conducted with a convenience sample of people living with INOCA. Participants were aged 18 or over, based in the UK, and had a diagnosis of INOCA. This was defined as either microvascular angina, coronary microvascular dysfunction, vasospastic angina, coronary vasospasms, coronary artery spasms, Prinzmetal/variant angina, angina with no obstructive coronary artery/INOCA. Guided by previous research detailing the challenges of diagnosis, and advice from a patient with lived experience of INOCA, we did not require participants to provide evidence of a definitive diagnosis but invited them to self-identify based on their current medical advice. Ethical approval was granted by Sheffield Hallam University (Ref: ER39732717).

A brief study advert (online supplemental information 2) providing information about the study was distributed via online patient support groups, social media and the British Heart Foundation ‘Heart Voices’ network. People expressing an interest contacted the lead researcher who provided a written study information sheet, by email or post, and followed up 24 hours later to discuss the study. For those who met the eligibility criteria and wished to proceed, an interview was arranged via telephone or video conferencing (Microsoft Teams or Zoom). A consent form was provided for participants to complete and return prior to the interview.

Theoretical saturation did not apply in this study.^12^ Instead, the recruitment goal was based on the availability of participants and funding for researcher time. Recruitment ceased when all potential participants expressing an interest in the study advert had been given adequate time to respond and take part. A Standards for Reporting Qualitative Research checklist is provided.

### Data collection

It was anticipated that having never attended cardiac rehabilitation before, some participants may have limited knowledge of the scope of comprehensive cardiac rehabilitation, beyond the exercise-based component. A workbook (online supplemental material 1) was designed with guidance from a person living with INOCA to invite participants to reflect on their rehabilitation needs and outline standard cardiac rehabilitation content. Workbooks were sent to participants prior to the interview to read and make notes, although it was emphasised that this was optional. The interviewer was an experienced, female qualitative researcher with a PhD in health and exercise psychology. The workbook formed a semistructured interview guide, with prompts as appropriate to stimulate discussion. For example, the researcher invited participants to discuss their views towards attending cardiac rehabilitation; how they currently managed different aspects of their life (eg, diet, exercise, medications), what support (if any) they needed and how they felt current cardiac rehabilitation might meet those needs (or not). Participants were encouraged to discuss the areas of the workbook they felt were relevant to them and any other factors that might influence their attendance at cardiac rehabilitation. Interviews lasted a maximum of 1 hour and were audio recorded. Recordings were sent to a professional company via secure link for transcription and were anonymised.

### Data analysis

Reflexive thematic analysis with inductive, semantic coding^13^ was used to analyse the data. Two researchers, both with postgraduate psychology qualifications and experience in qualitative analysis reviewed half of the transcripts each. Initial themes were developed independently, and then compared, merged and refined. Further discussion with a third researcher supported the sense-checking of final themes. Preliminary themes with descriptions and example anonymised quotes were also shared with all interview participants as a form of member checking. Respondents agreed that the draft findings accurately reflected their views.

### Findings

17 people living with INOCA were interviewed (88% female; age range 31–69). Participant characteristics are provided in table 1:

**Table 1:** Participant characteristics

Characteristic	N
Participants recruited	17
Gender	
Male	2
Female	15
Age	
31–40	2
41–50	2
51–60	9
61–70	4
Self-reported diagnosis	
Coronary vasospastic angina (CVA)	1
Microvascular angina+suspected vasospastic angina (MVA+VSA)	1
Coronary artery spasm (CAS)	4
Microvascular angina (MVA)	5
Vasospastic angina (VSA)	1
Coronary microvascular dysfunction+coronary artery spasm (CMD+CAS)	1
Vasospastic angina/coronary artery spasm (VSA/CAS)	1
Microvascular angina+vasospasms (MVA+vasospasms)	1
Variant angina	1
Previous experience of cardiac rehabilitation	
Previously attended cardiac rehabilitation	7
Not previously attended	10

Five themes were generated:

1. Precursors to offering cardiac rehabilitation to people with INOCA.
2. Tailoring invitations to cardiac rehabilitation for people with INOCA.
3. Adjustments to core cardiac rehabilitation content for people with INOCA.
4. New or specific cardiac rehabilitation resources for people with INOCA.
5. Patient-centred outcomes from taking part in cardiac rehabilitation.

**Figure 1:**
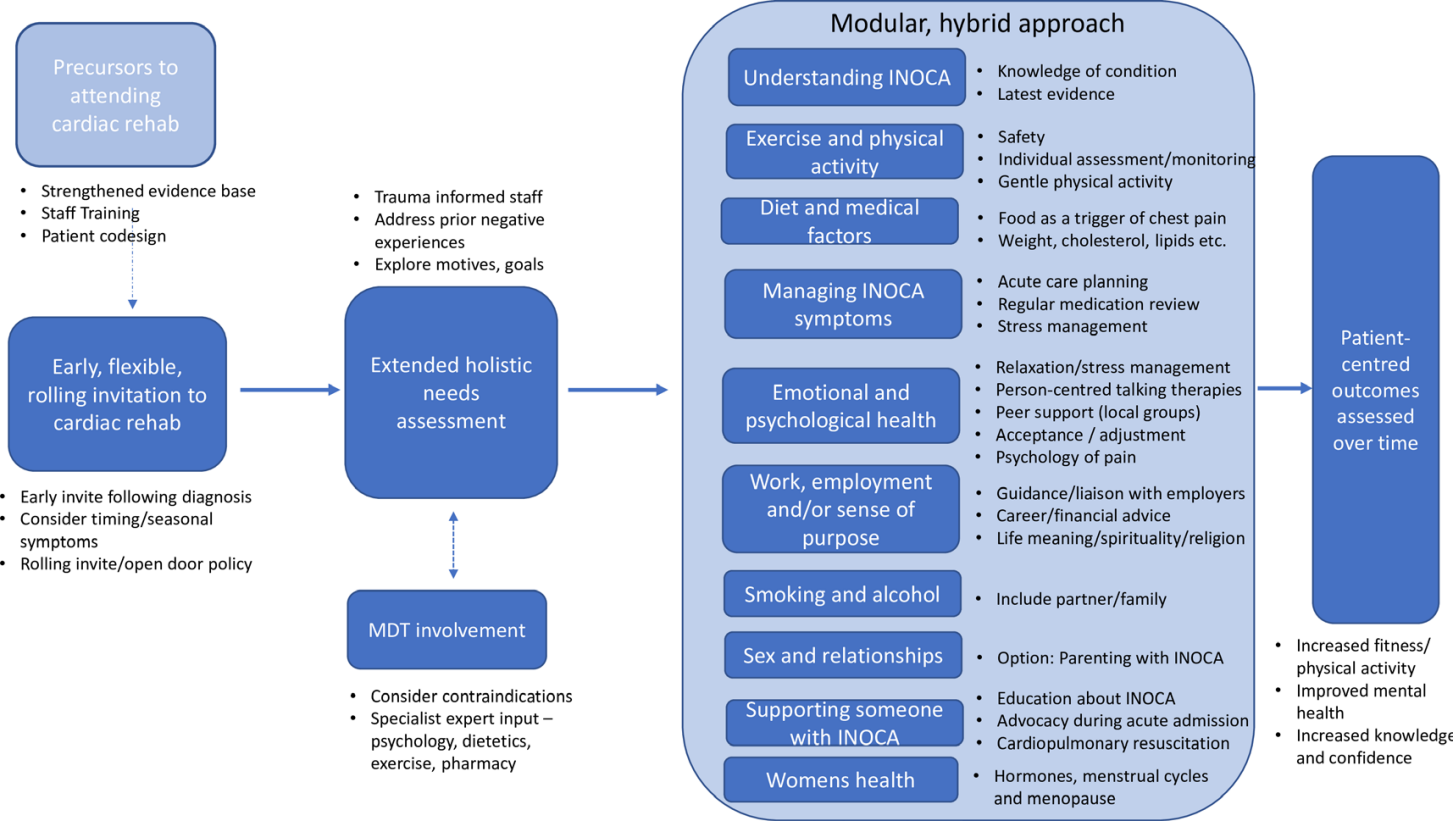
Final themes and key findings. CPR, cardiopulmonary resuscitation; INOCA, ischaemia with no obstructive coronary artery.

Figure 1 illustrates how these themes sit together. Each theme is described below along with illustrative participant quotes.

### Theme 1: precursors to offering cardiac rehabilitation to people with INOCA

Interviews highlighted concerns from people living with INOCA that need to be addressed prior to offering comprehensive cardiac rehabilitation for this group.

#### Staff training

Most participants reported concern and frustration about the lack of knowledge about their health condition among cardiac professionals, and examples were provided of encounters where healthcare professionals lacked detailed knowledge about INOCA:

… after that bad spasm last year, I was referred two days later to go and see the cardiac nurse specialist. She was telling me I needed to go on beta blockers. And I looked at her, I said that’s contraindicated for people with my type of angina, and she was a specialist cardiac nurse. And this isn’t that long ago, so there’s an awful lot more training that needs to be done with the medical profession. (P11; VSA/CAS)

Participants (regardless of prior cardiac rehabilitation attendance) suggested that specialist training for cardiac rehabilitation nurses and other professionals who are responsible for supporting patients with INOCA is needed.

#### Evidence-based rehabilitation guidance

In addition to staff training, some participants voiced concerns about the apparent lack of INOCA-specific research underpinning current cardiac rehabilitation:

Ultimately if the research is not there to provide the advice on optimal physical or diet or mental and emotional [support for people with INOCA], if they don’t have the research there it’s all going to be generic… (P2; MVA/VSA)

Previous attendees of cardiac rehabilitation shared other participants’ views regarding the need for staff training and an improved evidence base to support people with INOCA, and the need for adjustments to cardiac rehabilitation content detailed further in themes 3 and 4.

#### Patient-informed programmes

The involvement of patients with lived experience was highlighted as critical to tailoring cardiac rehabilitation appropriately. Recommendations were that the programme content, materials and evaluation measures were codesigned with people living with INOCA.

It’s not just a case of oh look, we spoke to a bunch of women with microvascular angina, and they would like cardiac rehab so let’s go and do it…the people that are doing the clinics need to…understand and talk to [people with INOCA] before you go away and set them up (P15, MVA).

### Theme 2: tailoring invitations to cardiac rehabilitation for people with INOCA

Participants who had previously taken part in cardiac rehabilitation generally believed it could be beneficial for others and supported the notion of increasing accessibility for people with INOCA. They typically recommended that people with INOCA should be referred at the earliest opportunity following a diagnosis; however, they also shared other participants’ views regarding the need for more flexible access, and some felt that they would like the opportunity to participate again, given the relapsing nature of their condition:

You can be fine for a while and then suddenly you’re in a flare up and having a really bad episode. For me they can last a couple of months, so after that your exercise capacity is back to zero. So it’s something that should be flexible to access because there are things on [cardiac rehabilitation] that I don’t need to access, like smoking cessation… But that exercise and that mental support and confidence building after an event is really important. (P2; MVA/VSA)

Potential challenges associated with INOCA diagnosis, and thus, early identification of eligible patients were raised:

This is difficult because if you’ve got somebody going into A&E…how do you find them? How do you get referred? The hospital that I’m at, the only people who get cardiac rehab are people who have had angioplasties, surgery, heart attacks, heart failure, and everybody else doesn’t… (P1, CMD).

It was suggested that the variable nature of the condition would affect responses to a cardiac rehabilitation invitation and thus programme delivery needed to be flexible. For example, cold weather was a common trigger for INOCA symptoms, meaning that many people would prefer online delivery during the winter months. Alternatively, they may not be able to complete exercise-based rehabilitation during winter but their needs for emotional support at that time might be greater.

A modular approach, enabling the creation of a personalised cardiac rehabilitation programme was preferred. Participants described different levels of knowledge about their triggers and patterns of symptoms, suggesting a need for extended assessment periods for some patients. It was also suggested that a multidisciplinary approach is necessary to ensure personal cardiac rehabilitation plans incorporate other comorbidities.

### Theme 3: adjustments to cardiac rehabilitation content for people with INOCA

Participants’ views suggest that most elements of existing cardiac rehabilitation programmes are relevant, provided that adjustments are made to acknowledge specific INOCA-related considerations.

#### Emotional/psychological health

Emotional and psychological health was highlighted by all participants as a vital component of cardiac rehabilitation, but those who had previously attended cardiac rehabilitation suggested that this element had not met their needs fully. Some recommended referral to talking therapy with a therapist who understood INOCA:

… [in] an ideal world, if there could be a counsellor who’s aware of how a condition like this would impact someone, I do think that would be helpful, and just give you coping strategies of how to get through that dark bit at first. (P4; CAS)

In many cases, this emotional support would be a gateway to engagement in other parts of cardiac rehabilitation, particularly exercise:

… to understand that person’s journey to that diagnosis and how they were made to feel by the system, because I think as well if your symptoms are brought on by exercise trust is huge, and if your experiences of the system have led to a lack of trust… why would you go along to cardiac rehab and trust that these people are going to keep you safe, and these people are going to understand your journey and your fears? (P15, MVA)

Managing emotions and stress long term was regarded by almost all participants as integral to self-management. Mindfulness approaches, boundary-setting and anxiety management were discussed. Participants also suggested that talking to people with shared lived experiences carried numerous benefits for emotional well-being and many expressed a desire for local in-person peer support:

I suspect, for me it’s support of others… it would be the emotional support with people that also experience it, which is why I joined the [online support] group, but they’re still anonymous faces. I would like to see a support group set up locally. (P11; VSA/CAD)

#### Exercise and physical activity

Participants varied in their attitudes towards exercise-based rehabilitation, but safety was a universal concern. Fear of pain and exacerbation was a significant barrier, and some (but not all) participants who had previously attended cardiac rehabilitation had found the exercise too demanding or triggering chest pain. Most participants would prefer face-to-face exercise support to provide reassurance, including support to establish a safe exercise baseline and tailored exercise plan. This would ideally be undertaken by an exercise professional with specific knowledge of INOCA who understood how INOCA symptoms impacted functional status episodically:

So I am quite worried about the exercise part of it because… I’ve only been dealing with this for a year, I’m not at a point yet where I know where my cut-off is… because it is a very fine line between too much and too little… (P9; CAS).

Some participants expressed disinterest in gym-based, circuit-style exercise which they associated with standard cardiac rehabilitation, stating that it was not female-friendly or was too removed from the physical activities they enjoyed (eg, swimming, walking). Several participants expressed a need for gentler forms of physical activity (eg, yoga, tai chi) that felt more achievable and could simultaneously address their needs around relaxation and stress management.

#### Work, employment and/or sense of purpose

Participants described varying experiences of employment since the onset of their symptoms. Many had been forced to give up work because of their condition and desired specialist vocational advice which covered access to financial benefits but also adjusting to life and identity without work:

Career adviser, I think it should be a very important part of rehabilitation… It’s not just about earning money and have enough money…Advice, for me, is the focusing on a purpose to live. (P13; CMD)

Participants who were still working suggested a need for written information suitable for employers that could explain their condition and advocate for reasonable adjustments at work:

I think it would be good to have an information booklet for employers. Because there’s one for rheumatoid arthritis, which I gave my employers, but I don’t know of any and haven’t been given any by my cardiologist… when I have an episode at work they always go into panic mode. (P6; VSA)

#### Diet

Some participants identified food as a trigger for chest pain attacks (eg, reacting to specific foods or overeating) and most avoided alcohol for similar reasons. Several participants followed dietary guidance published by UK health charities but suggested that INOCA-specific dietary advice should be incorporated into cardiac rehabilitation:

If I don’t watch my portion control I get spasms. If I eat something that’s got high inflammatory properties, I’m going to get spasms. If I’m hungry or skip meals I’m going to get spasms…You really have to probe down into the kinds of dietary advice when it comes to microvascular angina… and that requires specialist input (P2; MVA/VSA).

#### Medical management

Participants reported a significant amount of trial and error with medicines which could be confusing and daunting. They suggested a desire for a regular review and more education regarding pharmacological treatment:

A review of the medication I’m on primarily based from the point of view of the cardiac side. Because I get it for the diabetes side every year when I have a review; I don’t get anything for the cardiac side at all. (P12; CAS)

### Theme 4: new or specific cardiac rehabilitation resources for INOCA

In addition to standard cardiac rehabilitation topics, participants indicated other areas that would benefit from specialist modules designed for people living with INOCA:

#### Relationships and support for friends and family

Participants recognised that sex could be a trigger for chest pain. They felt that discussion of this topic should be an option in cardiac rehabilitation, but none recalled having this previously. It was suggested that family and friends could benefit from information and support, with topics including helping someone with INOCA to manage everyday activities, advocating for them during acute in-patient admissions and how to perform cardiopulmonary resuscitation:

… more information for family and friends… I think if [my partner] had something that was in black and white, written by a professional, he would take his time to understand that and maybe it would start a conversation of ‘oh is this what you go through, what can I do to help more’? (P14; MVA)

#### Female-specific issues

Several female participants expressed a desire to understand the link between their condition and hormones, menstruation and/or menopause. For example, one participant explained that menopausal symptoms could trigger her spasms, and others wanted to better understand why women are disproportionately affected by INOCA and the most recent evidence underlying female pathophysiology:

… the other thing to think about exercise is that the spasms can be altered by which period you are in your menstrual cycle, the hormones, so there’s things like that. So there’s lots of things that are women bound, hormones plus fertility… Mirena coils are useful because they stop your periods and it helps reduce the hormonal fluctuation…and yeah, how heart disease affects women differently. (P1; CVA)

…of course [menopause] makes [my spasms] worse as well. Yeah I get very menopausal. That’s when they speed up. (P12; CAS)

Additionally, one participant commented that women-only cardiac rehabilitation was needed to allow some women to participate according to their religious or cultural needs, but could also successfully bring together patients with INOCA due to the higher prevalence among women:

….(some) women for religious reasons or cultural reasons would not attend with men… And you’d probably find if you do have a woman’s only [cardiac rehabilitation] you’re more likely to get people with… microvascular angina… together because predominantly it affects women (P1; CMD).

#### Acute care planning

Participants wanted clear, personally tailored plans for managing acute symptoms. This could include managing chest pain at home, knowing when to seek medical intervention and having a cardiologist-approved, written care plan covering clinical and nursing care that they could present on admittance to hospital:

… sort of when to call an ambulance… maybe having an admission plan around that, and having it written down and saying this is why I’m presenting, I’m following the plan that I’ve been given, that would be helpful. (P6; VSA)

### Theme 5: patient-centred outcomes from taking part in cardiac rehabilitation

A number of participants emphasised the ongoing and changeable nature of their condition and that outcomes might therefore need to be measured over a longer period of time:

… outcome measures are difficult, because if you try to measure my outcome on a good day you’ll get a different measure to a less good day. So you’ve got to look longer term probably. (P16, variant angina)

Participants were asked to consider what would constitute a meaningful outcome for them, if they were to take part in cardiac rehabilitation. Many participants would like to have improved fitness and increased self-efficacy for daily physical activity such as walking and gentle exercise:

I think if I felt then at the end… more confident and was in a healthy enough place to increase some of my activities, so I could go back to swimming and I knew how to manage that (P7; MVA).

For many participants, physical improvements in fitness or energy and the confidence to be active would have mental health benefits:

I think having more confidence to do more things, like go walking maybe on my own and not having to depend on people, so it would help my mental health in that respect. (P4; CAS)

Ultimately, participants wished for increased knowledge about their condition and to feel more able to manage its ongoing impact on their lives:

just being better informed about what I could do to help myself in the best way. (P7; MVA)

## Discussion

Previous research involving patients with other cardiac conditions has highlighted broadly similar motivations for attending or declining cardiac rehabilitation to those suggested by INOCA patients in the current study. Cardiac rehabilitation was regarded by our participants and those in previous research as a way to reduce symptoms and increase optimism for the future.^14^ However, alongside this motivation, there is a strong need for positive beliefs about safety and effectiveness and a need for timely and accessible invitations into cardiac rehabilitation.^15^ Except smoking cessation, most topics covered within standard cardiac rehabilitation were considered relevant for INOCA patients.

### Psychosocial support

Previous research involving other cardiac patient groups has found that participants are dismissive of typical psychological techniques used in cardiac rehabilitation and reluctant to discuss their concerns with cardiac rehabilitation staff^16^ while facilitators have acknowledged limitations in the psychological support offered.^17^ Most participants in the current study would welcome a referral to qualified therapists but were also open to discussing their anxieties with cardiac rehabilitation staff, recommending that they were trained in trauma-informed practice. Psychological health was considered such a fundamental part of rehabilitation that it was clear that for many participants this was a vital ‘first step’ in cardiac rehabilitation which could facilitate engagement in other components. Participants consistently reported that stress management was also crucial to self-management of INOCA, supporting previous research highlighting mental stress as the most common trigger of angina in INOCA patients.^18^ Meditation and other stress reduction techniques are recommended for treatment and management of INOCA^19^ but few studies have explored the relative effectiveness of specific techniques.^20^ Participants in the current study suggested a wide range of potential useful approaches, indicating more research is needed to inform best practice.

### Evidence and expertise to inform lifestyle components

Anxiety is common among cardiac patients regardless of specific diagnosis, particularly among those who have experienced acute cardiac events, and there is a particular fear concerning exercise.^21^,^23^ Paradoxically, studies have found that many participants in comprehensive cardiac rehabilitation perceive the exercise component of their programme as the most significant contributing factor in health and well-being improvements.^16 24^ Trust in the expertise of professionals has been identified as an important prerequisite for engagement in cardiac rehabilitation, and previous research has identified the important role of allied health professionals such as physiotherapists to provide ‘expert’ advice on elements such as exercise.^25^ Previous thematic synthesis of qualitative studies suggests that exercise tailored to an appropriate level, with adequate staff support and a positive experience of the exercise component are critical to adherence.^26^ Cardiac rehabilitation standards refer to best practice guidance for exercise prescription but there is currently a paucity of evidence regarding what is effective and safe for people with INOCA.^27^ In patients with coronary artery disease and coronary heart disease, programmes using gentle exercise such as tai chi have shown promising benefits for improving aerobic endurance and psychosocial well-being.^28^ The desire of participants in the current study for gentle and safe exercise options suggests further research is needed to explore the acceptability, cost-effectiveness and effectiveness of these approaches for disease risk reduction in INOCA patients. Similar research is needed to optimise dietary advice for people with INOCA. Our findings support previous calls for multidisciplinary approaches including pharmacists, psychologists, dieticians and other allied health professionals in developing and delivering these components.^29^

Qualitative systematic reviews have identified additional factors influencing cardiac rehabilitation attendance including physical or financial barriers (eg, transport, cost).^30 31^ These barriers were not commonly discussed by participants. Nevertheless, many highlighted a need for high quality information about INOCA for employers, and this should include a clear rationale for employer support to attend cardiac rehabilitation.

### Format and delivery of cardiac rehabilitation

Participants in the current study indicated a desire for a modular, individually tailored cardiac rehabilitation programme. These principles are reflected in recently updated standards for cardiac rehabilitation^8^ which propose a patient-centred, menu-based approach. More than half of participants had only a ‘presumed’ diagnosis at the time of the study and several were not under the care of cardiologists. Typical referral pathways to cardiac rehabilitation are based on the identification of eligible patients as in-patients after an acute cardiac event.^8^ INOCA patients may not be known to local cardiac rehabilitation teams if they are managed through primary care or seeking a diagnosis privately, which evidence suggests may be common.^11 18^ Participants stated that they would potentially feel unable to enter cardiac rehabilitation during winter months or times when episodic symptoms are particularly disabling, meaning that they could require repeated invites similar to guidance for heart failure patients.^8^. Some INOCA patients with unstable or variable symptoms will also need an extended assessment period and this may need to be revisited more than once.

Increasingly cardiac rehabilitation incorporates hybrid delivery models (ie, a combination of in-person and remote delivery).^32 33^ Flexible delivery formats are effective, cost-effective and feasible and have shown to have benefits for other cardiac patients,^34^ but little research has explored home-based cardiac rehabilitation for patients with INOCA.^20^ Delivering some specific INOCA components online could provide an opportunity to connect people with others with similar diagnoses across larger geographical locations. However, many participants craved face-to-face peer support, and services should explore how to facilitate this locally when offering hybrid cardiac rehabilitation.

### Women-specific cardiac rehabilitation

Women are disproportionately affected by INOCA^1^ and several expressed a desire for more information about female-specific issues, including menstruation and menopause. There have been calls for a more sex- and gender-sensitive approach to understanding and treating cardiovascular health^35 36^ and ongoing trials investigating sex differences in INOCA.^37^,^39^ Our findings support these calls for further research to develop female-specific information and support. A recent systematic review suggests that women-focused cardiac rehabilitation is offered in 40% of countries. Some of these programmes offer alternative exercise modalities and more than half have a psychosocial focus.^40^ More work is needed to improve the cost-effectiveness and potential reach of cardiac rehabilitation to enable women with INOCA and other cardiac diagnoses to participate.

### Limitations

In the current study, 17 out of 22 eligible people who enquired about the interviews went on to take part. We did not ask non-participants to provide a reason for declining, and thus cannot speculate about their reasons. There may be some bias in our sample towards those with an interest in cardiac rehabilitation participation, although we did recruit a mix of previous attenders and non-attenders. Diagnosis and treatment of INOCA remains complex and inconsistent,^19^ so it is difficult to comment on how representative our sample was of the global INOCA patient population. However, evidence shows that women are disproportionately affected and the patient group is typically younger than traditional myocardial infarction and CAD patients^41 42^ and this is reflected in our sample. We did not collect socioeconomic or geographical information, so are unable to speculate about how these factors may influence our findings. Nevertheless, our participants were recruited from online patient support groups, so they were digitally included. Challenges associated with definitive diagnosis of INOCA limit our conclusions about how the time since diagnosis might impact experiences or attitudes towards cardiac rehabilitation, but this could be important to consider in future codesign of programmes.

## Conclusion

Current cardiac rehabilitation models may not fully meet the needs of people with INOCA. Further work is needed in several areas:

1. Optimise referral pathways into cardiac rehabilitation for patients including guidance to support timely identification of people living with INOCA.
2. Develop evidence-based cardiac rehabilitation guidance specific to INOCA and codesigned with patients.
3. Develop enhanced emotional well-being and/or psychological support for people with INOCA. Consider women-only cardiac rehabilitation options to increase accessibility.
4. Training for cardiac rehabilitation professionals, codesigned with patients, in how to tailor rehabilitation support specifically for people with INOCA.
5. Provide flexible, modular cardiac rehabilitation that INOCA patients can access as and when they can or need.
6. Develop guidelines for conducting cardiac rehabilitation assessments for people with INOCA with longer assessment period due to relapsing/remitting symptoms and informing tailored cardiac rehabilitation plans.
7. Generate evidence on the effectiveness of low-impact physical activity options for INOCA.
8. Codesign acute admission care planning templates for people with INOCA.
9. Establish networks of locally organised, face-to-face peer support groups for people living with INOCA.

## supplementary material

10.1136/bmjopen-2024-086770 online supplemental file 1

10.1136/bmjopen-2024-086770 online supplemental file 2

## Data Availability

Data is not available.
