# Supplementary material for: Exploring patients’ views regarding the support and rehabilitation needs of people living with myocardial ischaemia and no obstructive coronary arteries: a qualitative interview study
Source: BMJ Open. 2024 Dec 10;14(12):e086770. doi: 10.1136/bmjopen-2024-086770 (PMC11647337; doi:10.1136/bmjopen-2024-086770)
Supplement: online supplemental file 1 [file bmjopen-14-12-s001.pdf]

Organisational logos removed for publication

Do you have a presumed or confirmed diagnosis of:

- Microvascular angina
- Coronary Microvascular dysfunction/disease
- Vasospastic angina
- Coronary Vasospasms
- Coronary Artery Spasms
- Prinzmetal /Variant Angina
- Angina/ischaemia with no obstructive coronary arteries (ANOCA/INOCA)

We are looking for up to 20 people to take part in a research study involving a 1-hour telephone interview with an experienced researcher.

The study aims to understand:

- what it is like to live with these conditions
- how you manage your symptoms
- how suitable an existing programme called cardiac rehabilitation (which is offered to people with other types of heart condition) might be for you.

CONTACT US

To find out more about the study or how to take part, please email:

Dr Helen Humphreys
h.humphreys@shu.ac.uk
