# Supplementary material for: Exploring patients’ views regarding the support and rehabilitation needs of people living with myocardial ischaemia and no obstructive coronary arteries: a qualitative interview study
Source: BMJ Open. 2024 Dec 10;14(12):e086770. doi: 10.1136/bmjopen-2024-086770 (PMC11647337; doi:10.1136/bmjopen-2024-086770)
Supplement: Supplementary file 3 [file bmjopen-2024-086770.reviewer_comments.pdf]

PEER REVIEW HISTORY

BMJ Open publishes all reviews undertaken for accepted manuscripts. Reviewers are asked to complete a checklist review form and are provided with free text boxes to elaborate on their assessment. These free text comments are reproduced below.

ARTICLE DETAILS

Title (Provisional)

Exploring patients’ views regarding the support and rehabilitation needs of people living with myocardial ischaemia and no obstructive coronary arteries: a qualitative interview study

Authors

Humphreys, Helen; Paddock, Danielle; Brown, Sarah; Cowie, Aynsley; Berry, Colin; Dawkes, Susan; Nichols, Simon

---

VERSION 1 - REVIEW

**Reviewer**	**1**
**Name**	**Wang, Xiyi**
**Affiliation**	**Shanghai Jiao Tong University School of Nursing**
**Date**	**15-May-2024**
**COI**	**None**

Thank you for inviting me to review this manuscript. The manuscript underscores the importance of cardiac prevention and rehabilitation among INOCA patient populations. However, there are notable deficiencies that need addressing.

Introduction:

1. The discussion on the patient journey should elaborate on the variances and commonalities among INOCA and other CVD patients, highlighting the indispensability of the CPRP program and its role as a treatment.

2. Page 4, lines 106-108: It's imperative to provide a reference to support the assertion made in this sentence.

3. Page 5, lines 115-119: A critical analysis of the theoretical or conceptual frameworks and study hypotheses of the two papers is warranted. Moreover, it's essential to distinguish between the lived experiences and perspectives on CPRP treatment, incorporating lifestyle factors, based on the respective research questions posed in each study.

Method:

1. Further elucidation on the sampling procedure, including discussions on data saturation and representativeness, is necessary.

2. The study design and findings should adhere to proper reporting guidelines and demonstrate in the main manuscript.

3. Additionally, more information regarding the interview outlines and how they guided the data analysis process, including any employed code tree or framework, should be provided to enhance methodological transparency.

Results

1. To bolster the credibility of the findings, it's advisable to replace ambiguous terms like "may" (page 8, line 189) with more definitive language.

2. For theme 3, specific suggestions for tailoring CPRP to target INOCA patients should be extracted, rather than maintaining commonly concerned subthemes. Similarly, theme 4 should offer more detailed exploration of female-specific issues, given the higher prevalence of INOCA in this population.

3. Additional information about patient-centered outcomes and their correlations with CPRP participation is warranted.

4. Summarizing the interview outline, coding framework, and study findings at a theoretical level would enhance the manuscript's applicability to broader social and cultural contexts.

Discussion:

1. While the study's focus is on the UK context, it would enrich the discussion to include international references and enhance global generalizability.

2. Furthermore, guideline recommendations for the application of CPRP to INOCA patients based on the study's findings should be delineated. Incorporating other relevant findings pertaining to INOCA patients would strengthen the discussion and provide avenues for future research directions.

3. Regarding page 17, line 413, additional information and evidence are needed to support the statement made.

Conclusion:

The conclusion lacks robust findings, particularly as patient evaluations of the CPRP program and details on CPRP delivery modes based on patient experiences are absent from the study. This aspect should be addressed to enhance the conclusiveness of the findings.

**Reviewer**	**2**
**Name**	**Mazzoni, Gianni**

Affiliation **University of Ferrara, Center of biomedical studies applied to sport**

Date **28-Jun-2024**

COI **Nothing to declare**

I would like to express my gratitude for the opportunity to review this paper and contribute to the ongoing discourse in this important area of cardiovascular care. This paper provides a compelling exploration into the experiences and perspectives of patients with myocardial ischaemia and no obstructive coronary arteries (INOCA) regarding their engagement with cardiovascular prevention and rehabilitation programmes (CPRP). The study is methodologically robust, employing semi-structured interviews and inductive thematic analysis to yield insightful data. The findings, which highlight the unique needs and preferences of INOCA patients, are particularly valuable for informing the design and delivery of more inclusive and effective rehabilitation support. However, there are certain areas within the paper that require further clarification and elaboration. Thus, while the paper is well-conceived and executed, a minor revision is necessary to address these points and enhance the overall clarity and impact of the study.

Abstract

The aim of the study is not clearly stated. In order to make the abstract more clear please clearly state what the purpose of the study is.

Results section looks like more a methods section since in this part is not reported what came out from the interview instead a list of main themes touched during the interview is reported. Please provide a summary of the results and move the current result section to the methods.

Line 115-119

Since the results related to the first aim of the study are not reported in this paper, it is counterintuitive to describe it as the first aim of the present study. It would be reasonable to report the findings related to the first aim of the study in the present paper. Otherwise, report the second aim of the study as the first aim.

Line 132

Please clarify how the study was advertised to avoid communicating researcher expectations or introducing selection bias.

Line 140-141

Since some participants did not take part in the Cardiac Pulmonary Rehabilitation Program (CPRP), it would be useful to:

1. Report in the results section how many participants had previously taken part in the CPRP.

2. Properly discuss the differences in experiences between participants living with INOCA who participated in the program and those who did not.

Line 151: It could be useful to provide a rationale for using this strategy and to clarify how the questions were developed according to this type of analysis.

Line 163: It could be useful to report the time elapsed from diagnosis of INOCA since experience and perceptions of participants could be impacted by this parameter

Line 191: It should be clarified if these kinds of answers were provided by participants previously involved in CPRP, in order to determine if this is a belief about CPRP or a lived experience.

Discussion

I recommend to add a limitation section in your discussion

Line 369-37: It could be useful to compare the experiences and perceptions of patients living with Ischemia and No Obstructive Coronary Artery Disease (INOCA) who are involved in cardiac rehabilitation with those of other cardiac patients. Additionally, the potential role of other professional figures in their care should be considered.

The results of this comparison should be discussed considering findings from other relevant studies.

For instance:

Raisi A, Piva T, Myers J, Zerbini V, Mandini S, Zappaterra T, Mazzoni G, Tonet E, Pavasini R, Campo G, et al. Experience and Perceptions among Older Outpatients after Myocardial Infarction following an Exercise Intervention: A Qualitative Analysis from the PIpELINe Trial. International Journal of Environmental Research and Public Health. 2023; 20(3):2196. https://doi.org/10.3390/ijerph20032196

Bäck, Maria, Birgitta Öberg, and Barbro Krevers. "Important aspects in relation to patients' attendance at exercise-based cardiac rehabilitation–facilitators, barriers and physiotherapist's role: a qualitative study." BMC cardiovascular disorders 17 (2017): 1-10.

VERSION 1 - AUTHOR RESPONSE

Comment	Response	Page/line number(s) (clean version)
**Reviewer 1**		
Introduction:<br>1. The discussion on the patient journey should elaborate on the	Additional references have been added comparing INOCA patients to CAD patients in terms of QoL,	Page 3-4 Lines 85-90

variances and commonalities among INOCA and other CVD patients, highlighting the indispensability of the CPRP program and its role as a treatment.	angina burden, risk of major cardiovascular event, etc.	
2. Page 4, lines 106-108: It's imperative to provide a reference to support the assertion made in this sentence	Unfortunately our page numbers do not appear to match with the line numbers referred to here but we have added the relevant reference where we think it's needed.	Page 4<br>Line 102
3. Page 5, lines 115-119: A critical analysis of the theoretical or conceptual frameworks and study hypotheses of the two papers is warranted. Moreover, it's essential to distinguish between the lived experiences and perspectives on CPRP treatment, incorporating lifestyle factors, based on the respective research questions posed in each study.	This section has been updated to clearly outline the research questions for the study and highlights its exploratory nature (i.e. no prior hypothesis or theory testing).	Page 5<br>117-129
Method:<br>1. Further elucidation on the sampling procedure, including discussions on data saturation and representativeness, is necessary.	We have added some additional detail regarding sampling and saturation.<br><br>We have added discussion regarding representativeness in the new Limitations section at the end of the Discussion.	Page 6<br>151-155
2. The study design and findings should adhere to proper reporting guidelines and demonstrate in the main manuscript.	We have provided an SQRQ checklist and referenced this in the text.	Page 6<br>155
3. Additionally, more information regarding the interview outlines and how they guided the data analysis process, including any employed code tree or framework, should be provided to enhance methodological transparency.	More information about how the current CPRP content was used to form an interview guide, inviting participants to comment on its appropriateness for their needs has been added.<br><br>Additional information regarding the process of inductive thematic analysis has also been added.	Pages 6-7<br>158-173
Results<br>1. To bolster the credibility of the findings, it's advisable to replace ambiguous terms like "may" (page 8, line 189) with more definitive language.	Thank you for highlighting this. We have replaced ambiguous terms such as "may be".	Various

2. For theme 3, specific suggestions for tailoring CPRP to target INOCA patients should be extracted, rather than maintaining commonly concerned subthemes.	This theme has been further developed to highlight more specific suggestions about tailoring CPRP made by participants.	Pages 12-14
. Similarly, theme 4 should offer more detailed exploration of female-specific issues, given the higher prevalence of INOCA in this population.	Additional explanation has been provided for Theme 4.	Page 15 351-370
3. Additional information about patient-centered outcomes and their correlations with CPRP participation is warranted.	Further detail has now been added to this theme with quotes to illustrate the more specific patient-centred outcomes suggested by participants.	Page 16 380-398
4. Summarizing the interview outline, coding framework, and study findings at a theoretical level would enhance the manuscript's applicability to broader social and cultural contexts.	As the study was intended to be inductive, we have added additional explanation within the methods section about how we used the UK CPRP standards to inform the interview guide, but allowed participants to discuss only those areas that felt relevant to them. We did not apply a specific theoretical framework. We have experimented with discussing our findings in light of a specific health psychology or sociological theory of illness, but have found this does not add substantially to the paper's value. Instead, we have added Figure 1, which provides a visual overview of the final themes and key findings, which we hope could be useful for others to reinterpret in light of other models of CPRP beyond the UK. We also now discuss the applicability or transferability of the findings to other contexts in our newly added limitations section of the discussion.	Page 9 197-199
Discussion:<br>1. While the study's focus is on the UK context, it would enrich the discussion to include international references and enhance global generalizability.	Additional international references have been added where suitable	Various
2. Furthermore, guideline recommendations for the application of CPRP to INOCA patients based on the study's findings should be delineated. Incorporating other relevant findings pertaining to INOCA patients would strengthen the discussion and provide avenues for future research directions.	Specific recommendations have been drawn out and used to strengthen the conclusion section.<br><br>Sadly, there is a dearth of previous research involving people with INOCA (which is partly the rationale for this study) but we have drawn on existing findings where possible throughout the discussion.	Page 21 502-521

3. Regarding page 17, line 413, additional information and evidence are needed to support the statement made.	The sub-theme describing female-specific issues has been expanded which hopefully addresses this point.	n/a
Conclusion:<br>The conclusion lacks robust findings, particularly as patient evaluations of the CPRP program and details on CPRP delivery modes based on patient experiences are absent from the study. This aspect should be addressed to enhance the conclusiveness of the findings.	Recommendations have been added to strengthen the conclusion.	Page 21<br>502-521

**Reviewer 2**		
**Comment**	**Response**	**Page and line numbers (clean version**
Abstract<br>The aim of the study is not clearly stated. In order to make the abstract more clear please clearly state what the purpose of the study is.	This has been updated to reflect the primary aims for the study.	Page 2<br>42-47
Results section looks like more a methods section since in this part is not reported what came out<br>from the interview instead a list of main themes touched during the interview is reported. Please<br>provide a summary of the results and move the current result section to the methods.	The results section of the abstract has been reworded to better summarise key findings.	Page 2<br>51-61
Line 115-119<br>Since the results related to the first aim of the study are not reported in this paper, it is counterintuitive to describe it as the first aim of the present study. It would be reasonable to report<br>the findings related to the first aim of the study in the present paper. Otherwise, report the second<br>aim of the study as the first aim.	This has been updated to reflect the primary aims of the current study and distinguish it from the secondary aim.	Page 5<br>117-129
Line 132<br>Please clarify how the study was advertised to avoid communicating researcher expectations or<br>introducing selection bias.	We have included reference to the study advert and now included this as Supplementary Information 2	Page 6<br>143

Line 140-141<br>Since some participants did not take part in the Cardiac Pulmonary Rehabilitation Program (CPRP),<br>it would be useful to:<br>1. Report in the results section how many participants had previously taken part in the CPRP.<br>2. Properly discuss the differences in experiences between participants living with INOCA who participated in the program and those who did not.	1. Figures summarising previous experiences of taking part in CPRP have been added to the results table.<br><br>2. An additional paragraph to address this point has been added under Theme 2. There were not major differences between these two groups of participants, and the themes presented reflect the views of both attenders and non-attenders. We have added nuance where appropriate to acknowledge this throughout the results section.	Page 8<br><br><br><br>Line 213<br>234-239<br>271<br>294-296<br>342-343
Line 151: It could be useful to provide a rationale for using this strategy and to clarify how the questions were developed according to this type of analysis.	We think this comment relates to the use of a workbook to guide the interviews (apologies if this is incorrect- our line numbers do not match). We have added more information about how current CPRP content was used to form an interview guide, inviting participants to comment on its appropriateness for their needs.	Pages 6-7<br>158-173
Line 163: It could be useful to report the time elapsed from diagnosis of INOCA since experience<br>and perceptions of participants could be impacted by this parameter	Unfortunately, we are unable to report this detail. As is the typical experience for many people living with INOCA, many of our participants did not have a confirmed diagnosis and were living with a presumed or suspected diagnosis without an explicit start date. Their experience of diagnosis thus spans the course of several years having potentially evolved depending on which cardiologist they see. The time from diagnosis is thus very unclear for many patients and cannot be accurately reported. We have acknowledged this in our newly added limitations section at the end of the discussion.	Page 20<br>497-500
Line 191: It should be clarified if these kinds of answers were provided by participants previously involved in CPRP, in order to determine if this is a belief about CPRP or a lived experience.	These views were shared by participants who had attended and those who had never attended CPRP - this been clarified in the text.	Various
Discussion<br>I recommend to add a limitation section in your discussion	Thank you. Limitations section now added.	Page 20<br>486-500

Line 369-37: It could be useful to compare the experiences and perceptions of patients living with<br>Ischemia and No Obstructive Coronary Artery Disease (INOCA) who are involved in cardiac rehabilitation with those of other cardiac patients.<br>The results of this comparison should be discussed considering findings from other relevant studies.<br>For instance:<br>Raisi A, Piva T, Myers J, Zerbini V, Mandini S, Zappaterra T, Mazzoni G, Tonet E, Pavasini R,<br>Campo G, et al. Experience and Perceptions among Older Outpatients after Myocardial Infarction<br>following an Exercise Intervention: A Qualitative Analysis from the PIpELINe Trial. International<br>Journal of Environmental Research and Public Health. 2023; 20(3):2196.<br>https://doi.org/10.3390/ijerph20032196<br>Bäck, Maria, Birgitta Öberg, and Barbro Krevers. "Important aspects in relation to patients'<br>attendance at exercise-based cardiac rehabilitation–facilitators, barriers and physiotherapist's<br>role: a qualitative study." BMC cardiovascular disorders 17 (2017): 1-10.	Additional references have been added and parallels drawn with other cardiac patient groups.	Various
Additionally, the potential role of other professional figures in their care should be considered.	A specific acknowledgement about the role of multidisciplinary teams has been provided.	Page 18<br>441-443

VERSION 2 - REVIEW

**Reviewer**	**2**
**Name**	**Mazzoni, Gianni**
**Affiliation**	**University of Ferrara, Center of biomedical studies applied to sport**
**Date**	**04-Nov-2024**
**COI**	

The authors have clarified all the issues outlined during the review process.
